# Group-based trajectory modeling of hemoglobin change rate and impact of haemoglobin concentration on cardiovascular outcome after endovascular aneurysm repair a risk prediction model for persistent postoperative hemoglobin decline

**DOI:** 10.3389/fragi.2026.1867588

**Published:** 2026-06-25

**Authors:** Xiao Li, Xiang Yan, Qing Sheng Lu

**Affiliations:** The Department of Vascular Surgery, The First Affiliated Hospital of Naval Medical University, Shanghai, China

**Keywords:** EVAR, hemoglobin decline, longitudinal cohort, nomogram, trajectory

## Abstract

**Objective:**

While postoperative hemoglobin decline following Endovascular Aneurysm Repair (EVAR) is a well-known phenomenon, the specific factors driving persistent hemoglobin reduction remain unclear. This observational, retrospective study seeks to establish distinct trajectories of hemoglobin change rates within the first 7 days following Endovascular Aneurysm Repair (EVAR) and to analyze the factors influencing persistent post-operative hemoglobin decline as well as Hb decrease, defined as preoperative minus postoperative Hb (g dl ^-1^), on postoperative cardiovascular events in EVAR patients.

**Methods:**

The study population consisted of all patients who underwent endovascular aneurysm repair (EVAR) for abdominal aortic aneurysm (AAA), totaling 1,995 individuals, between 1 June 2002, and 31 March 2024. Group-based trajectory modeling (GBTM) was utilized to categorize distinct patterns of hemoglobin changes over a 7-day period in patients following EVAR. Baseline clinical and demographic characteristics predictive of trajectory group membership were analyzed using univariate screening and two multivariate binary logistic regression. The development of the two risk prediction models for persistent postoperative hemoglobin decline in patients undergoing EVAR was undertaken using logistic regression. The corresponding nomograms were subsequently constructed. The study second endpoint was 30-day postoperative cardiovascular events, including. myocardial infarction, heart failure, arrhythmias, stroke, asymptomatic troponin-T release and cardiovascular death.

**Results:**

We identified two trajectory groups: ' The decrease surpasses 20%' (n = 251, 16.8%) and ‘under 20%' (n = 1,528, 83.2%). After adjusting for potential confounders, these groups were significantly linked to clinical outcomes like age, PAD, aneurysm characteristics. Intraoperative factors such as operation duration, blood loss, and graft details also showed associations. A risk prediction model, visualized with a nomogram, included factors like age, PAD, a larger maximum AAA diameter, and Iliac artery aneurysm presenting with bilateral involvement, achieving an AUC of 0.733 (95% CI: 0.690–0.775). The model showed 70.0% sensitivity and 66.7% specificity, with a calibration chart confirming strong alignment between observed and predicted values, and a clinical decision curve highlighting its potential clinical utility.

**Conclusion:**

In conclusion, our study identified that older age, maximum diameter of abdominal aortic aneurysm, and Iliac artery aneurysm presenting with bilateral were associated with continued hemoglobin decline post-EVAR. A drop in hemoglobin concentration exceeding 20% after elective EVAR surgery strongly predicts cardiovascular events within 30 days.

## Introduction

Abdominal aortic aneurysm is common in individuals over 60, with rupture risk increasing as the aneurysm grows. Endovascular aneurysm repair (EVAR) is the preferred treatment for infrarenal aneurysms, offering lower early mortality than open surgery (Patel et al., 2016). Post-implantation syndrome (PIS), identified by Velazquez et al., in 1999 (Velázquez et al., 1999), is an acute inflammatory response following EVAR with endograft placement (D'Oria et al., 2024; Ribeiro et al., 2023). In clinical practice, some patients experience post-implantation syndrome (PIS) along with acute anemia or coagulation issues, marked by decreased hemoglobin and platelet levels. In 2015, Lu Qing sheng et al. identified this as post-endovascular aortic repair syndrome (PERS), characterized by systemic inflammation and hemostatic imbalance after endovascular aortic aneurysm repair (EVAR). PERS involves a non-hemorrhagic decline in hemoglobin and platelet levels, alongside coagulation dysfunction. Changes in hemoglobin levels post-EVAR are crucial indicators of potential complications. Current guidelines inadequately estimate hemoglobin reduction after EVAR (Wanhainen et al., 2024; Blecha et al., 2022), potentially leading to postoperative complications, especially in women and those with preoperative renal issues. There is a lack of systematic analysis and recommendations for managing hemoglobin changes post-EVAR (Blecha et al., 2022).

This paper explores hemoglobin change rate trajectories post-EVAR using Group-based trajectory modeling (GBTM) (Nagin and Odgers, 2010), an unsupervised method that identifies distinct trajectory groups over time (Nagin, 2014). GBTM, a type of finite mixture modeling, is widely used in medicine and psychology to analyze longitudinal data (Nagin and Tremblay, 2001). This study is the first to apply GBTM to hemoglobin changes after EVAR, aiming to identify natural phenotypes more accurately than subjective clinical diagnoses. The different trajectory groups will be further analyzed to explore how demographic and clinical factors influence patterns of hemoglobin change and their associated risks.

## Materials and methods

### Study design and setting

**FIGURE 1:**
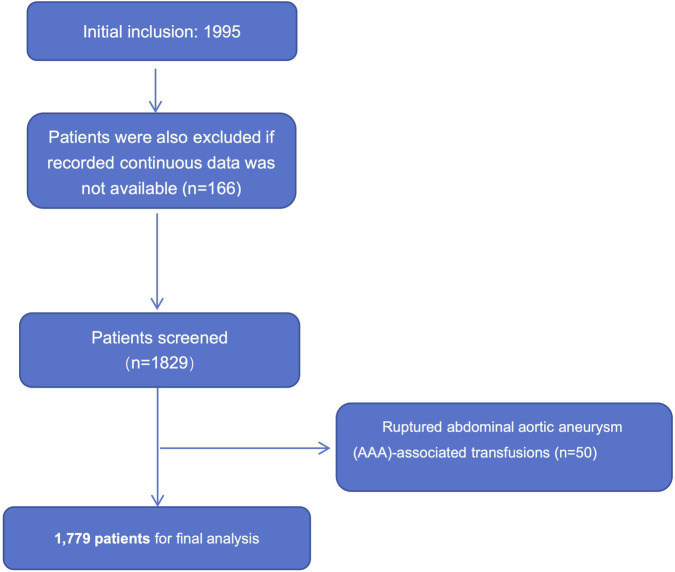
Study participants flow diagram.

This retrospective cohort study analyzed data from 1,995 AAA patients treated with EVAR at our university vascular surgery center between January 2002 and March 2024. Informed consent was waived because of the retrospective nature of the study and the analysis used anonymous clinical data. This study was waived from ethical review by the Institutional Review Board of Shanghai Changhai Hospital Medical Ethics Committee due to its retrospective design. The study adhered to the STROBE guidelines for cohort studies (von Elm et al., 2007). Patients for whom pre- or postoperative Hb concentrations were not available were excluded. The predefined exclusion criteria were as follows: lack of postoperative hemoglobin monitoring; absence of preoperative baseline hemoglobin levels; ruptured abdominal aortic aneurysm (AAA). In total, 216 participants (10.8%) were excluded, resulting in 1,779 eligible cases for analysis. The research flowchart is shown in Figure 1.

Upon hospital admission, patients underwent a detailed medical history review focusing on AAA risk factors, comorbidities, and medication. A comprehensive vascular exam and routine lab tests. Imaging involved iodine contrast-enhanced CTA for aortoiliac angiography, with anatomical data processed for preoperative planning using 3D CTA analysis software. Patients typically underwent a standard EVAR procedure, and daily blood samples were taken postoperatively to monitor hemoglobin levels. The study aimed to analyze hemoglobin changes within 7 days after EVAR.

### Group-based trajectory modeling and statistical analysis

Our study explored hemoglobin level changes in the first week after EVAR using a group-based trajectory modeling (GBTM). We used Stata 18s TRAJ package to classify participants into subgroups with similar hemoglobin change patterns. We chose the model with the highest Bayesian information criterion (BIC) and a sample size of at least 5% per trajectory group (Nguena Nguefack et al., 2020). Model adequacy was confirmed by: (1) an average group posterior probability over 0.7, (2) odds of correct classification of 5 or more, and (3) alignment between estimated group probabilities and group assignment proportions (Nagin and Odgers, 2010). Clinical relevance and plausibility of trajectory classes were also evaluated.

Two multivariable logistic regression models were developed to predict trajectory group membership. Potential predictors comprised demographic characteristics, anatomical features of the abdominal aortic aneurysm (AAA)—such as length, volume, and maximum diameter—and intraoperative factors. Univariate analyses were performed using the chi-square test and the Mann–Whitney U test. Variables with P < 0.05 in univariate analysis were included in multivariate binary logistic regression models to identify significant predictors, using a stepwise approach. Odds ratios (OR) and 95% confidence intervals (CI) were reported. To control for potential confounding, age, sex, hypertension, and diabetes were included as covariates in the final models. Analyses were performed using IBM SPSS (v27.0), with P < 0.05 considered statistically significant.

Finally, the significant variables identified from the logistic models were incorporated into two separate risk prediction models. Corresponding nomograms were generated to provide individualized risk scores, and the contribution of each independent risk factor was quantified using RStudio (v2024.10.31) and the rms package. The model’s predictive power was evaluated using the ROC curve and AUC, with a higher AUC indicating better prediction. Youden’s index determined the optimal clinical threshold, while sensitivity, specificity, PPV, and NPV were derived from the ROC curve. The Hosmer-Lemeshow test assessed model calibration, with a P-value > 0.05 indicating good fit. The C-index, close to 1, indicated strong calibration. Decision curve analysis evaluated clinical utility by comparing net benefits across risk thresholds. The study also examined how varying rates of hemoglobin change affect cardiovascular outcomes within 30 days post-surgery.

## Results

### GBTM of the hemoglobin change rate

**FIGURE 2:**
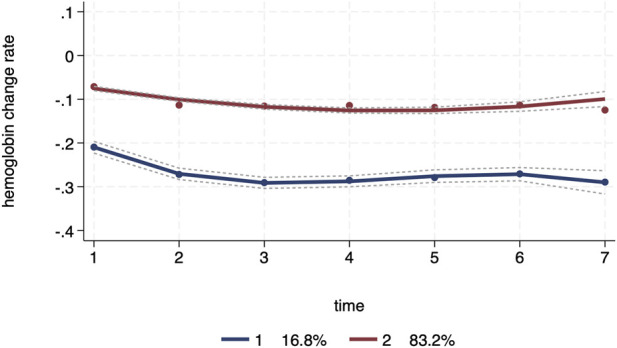
Two distinct trajectories of Hemoglobin change rate after EVAR.

Among the total of 1,995 participants in screening 1,779participants (89.2% men) ultimately entered this study (Figure 1). The BIC was lowest for the model with two trajectories (BIC = 4,337.79) and an entropy value of 0.766; moreover, the average posterior probabilities were more than 0.7 for two trajectories groups and only the 2-group model achieved the minimum group size of at least 5% for each group and was thus selected for further analysis. Thus, we identified the GBTM model with two trajectories as the optimal model. Figure 2 illustrates the longitudinal hemoglobin change rate patterns for two distinct trajectories observed after EVAR. Group 1, labeled’ The decrease surpasses 20%' (n = 251, 16.8%), exhibited a continuous decline over time, maintaining persistently low levels. Group 2, labeled ‘under 20%' (n = 1,528, 83.2%), showed minimal fluctuations throughout the 7-day postoperative period, maintaining consistently high levels.

### Trajectory sub-population characteristics

Table 1 outlines the baseline characteristics of participants in each trajectory group. The “The decrease surpasses 20%” group tended to be older, female, and have coronary artery and peripheral arterial occlusive diseases. Baseline preoperative blood tests showed no significant differences. However, the “The decrease surpasses 20%” group experienced longer operations (p < 0.001), more intraoperative blood loss (p < 0.001), more grafts (p = 0.001), and longer graft lengths (p < 0.001). Table 2 compares anatomical parameters of abdominal aortic aneurysms (AAA) between the hemoglobin decreasing surpasses 20% group (n = 1) and the hemoglobin decreasing under 20%group (n = 2), revealing significantly longer AAA lengths in Group 1 (111.5 mm [73–147] vs. 82 mm [61–113], P < 0.001). Group 1 had a larger mean AAA volume (72 ml vs. 58 ml, P < 0.001), shorter proximal neck lengths (31 mm vs. 36 mm, P < 0.001), and a larger maximum AAA diameter 52.6 mm [44.1–70.1] vs. 46.8 mm [38.2–58.2] compared to Group 2. There was no significant difference in the maximum true lumen diameter between the groups (33.5 mm vs. 33.5 mm, P = 0.079).

**TABLE 1:** The baseline characteristics of participants in each trajectory group.

Variables	The decrease surpasses 20% (1)	The decrease under 20% (2)	p
N	251	1,528	​
Age, years	74 (69–79)	71 (65–77)	<0.001
Sex, n (%),male	194 (77.3)	1,296 (85.8)	0.003
Smoking, n (%)	83 (33.1)	690 (45.2)	<0.001
Comorbidities			
Diabetes, %	30 (12.0)	224 (14.7)	0.246
Renal insufficiency, %	15 (6.0)	65 (4.3)	0.241
COPD, %	9 (3.6)	63 (4.1)	0.689
Hypertension, %	170 (29.1)	1,045 (28.1)	0.835
Cerebrovascular disease, %	31 (12.4)	154 (10.0)	0.274
Coronary artery disease, %	67 (26.7)	324 (21.2)	0.052
Peripheral arterial occlusive disease, %	23 (9.2)	67 (4.4)	0.001
Hyperlipidemia, n (%)	6 (2.4)	34 (2.2)	0.870
Atrial fibrillation,n (%)	17 (4.6)	40 (3.2)	0.223
Preoperative blood tests			
Hb, g/dL	133 (123–141.5)	131 (119–143)	0.311
Platelets, 109/L	179 (148.5–222.5)	182 (148–225.0)	0.582
Intraoperative variables			
Duration of operation, min	180 (120–220)	110 (80–150)	<0.001
Blood loss, mL	200 (80–400)	50 (50–100)	<0.001
Number of grafts	3 (2–5)	3 (2–4)	0.001
Length of grafts	184.5 (161–214)	170 (154–190)	<0.001

**TABLE 2:** Univariable analysis of factors influencing Persistent Postoperative Hemoglobin Decline.

Variables	The decrease surpasses 20% (1)	The decrease under 20% (2)	p
Abdominal aortic aneurysm length,mm	122.5 (90–153)	84 (62–117)	<0.001
Abdominal aortic aneurysm volume,ml	82.5 (45–159)	59 (35–95)	<0.001
Proximal neck length of abdominal aortic aneurysm,mm	28 (15–44)	35 (24–47)	0.001
Abdominal aortic aneurysm neck angle,°	145.1 (123.6–158.30)	147.8 (131.4–158.90)	0.181
Maximum diameter of abdominal aortic aneurysm,mm	52.6 (44.1–70.1)	46.8 (38.2–58.2)	<0.001
Abdominal aortic aneurysm true lumen maximum diameter,mm	33.5 (28.5–45.25)	33.5 (27.6–40.3)	0.079
Thickness of mural thrombus in abdominal aortic aneurysm,mm	15.55 (8.15–25.5)	12.4 (7.3–18.8)	0.001
Abdominal aortic aneurysm thrombosis rate (<25 = 1,25–50 = 2,50–75 = 3,>75 = 4,100 = 5)	4 (3–5)	4 (3–5)	0.011
Calcification rate of abdominal aortic aneurysm cavity (<25 = 1,25–50 = 2,50–75 = 3,>75 = 4,100 = 5)	2 (1–3)	1 (1–3)	0.003
Iliac artery aneurysm presenting with bilateral involvement,%	63 (61.8)	322 (38.8)	<0.001

### Multivariate binary logistic regression analysis of “consistently declining” trajectory group membership

Table 3 showed that the association of “ The decrease surpasses 20%” trajectory group membership and preoperative factors. Patients without PAD (OR = 0.552, P = 0.004) and male patients (OR = 0.484, P = 0.001) had a significantly lower risk of belonging to the “ The decrease surpasses 20%” group. Among the preoperative factors, age, PAD, a larger maximum AAA diameter, and Iliac artery aneurysm presenting with bilateral involvement as significant risk factors contributing to the likelihood of membership in the “ The decrease surpasses 20%” trajectory group.

**TABLE 3:** Multivariate analysis of preoperative factors.

Variables	B	OR (95% CI)	p value
Age	0.035	1.035 (1.006–1.065)	0.018
Absence of PAD	−0.860	0.42 (0.188–0.953)	0.038
Maximum diameter of abdominal aortic aneurysm,mm	0.020	1.020 (1.005–1.036)	0.009
Absence of iliac artery aneurysm	−0.801	0.44 (0.280–0.718)	0.001

### Construction of nomogram-based risk prediction models

**FIGURE 3:**
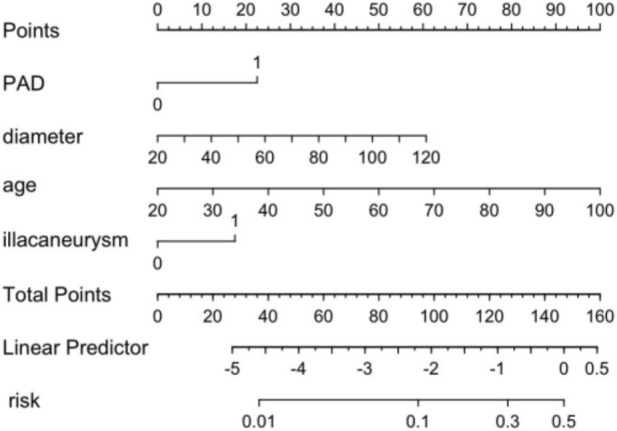
Nomogram-based risk prediction model for persistent hemoglobin decline after EVAR (PAD, Peripheral Artery Disease).

Development of a nomogram-based risk prediction model for persistent hemoglobin decline after EVAR using preoperative factors (Figure 3) as predictors. The nomogram-based risk prediction model operates by allocating specific scores to each predictor variable along the “Points” axis. These individual scores are aggregated to yield a total score, exemplified by a cumulative score of 125 for a hypothetical patient. This total score is subsequently translated into a linear predictor value (LP) on the subsequent axis, which is then converted into the final risk probability, such as a 30% likelihood of persistent hemoglobin decline surpasses 20%.

**FIGURE 4:**
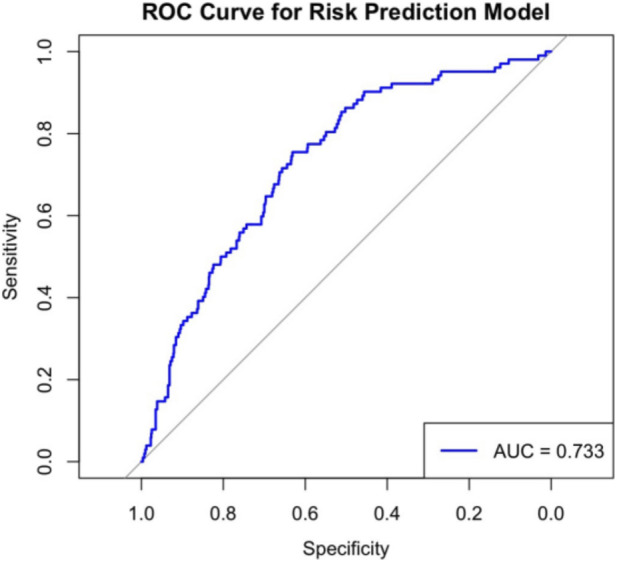
Evaluation of the nomogram prediction model of preoperative factors. ROC curve of the nomogram.

**FIGURE 5:**
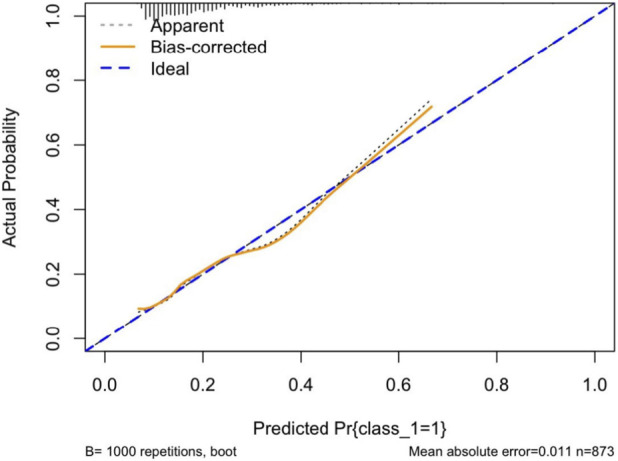
Evaluation of the nomogram prediction model of preoperative factors. Calibration graph of the nomogram.

**FIGURE 6:**
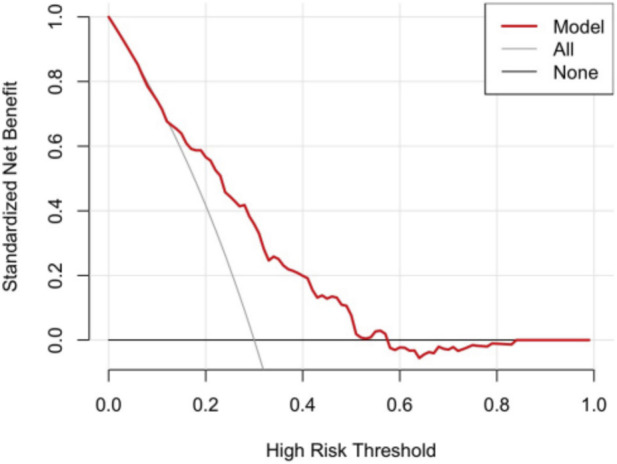
Evaluation of the nomogram prediction model of preoperative factors. Decision Curve Analysis of the nomogram.

The model’s effectiveness was assessed using ROC curves, calibration plots, decision curve analysis, sensitivity, and specificity. Initially, the model effectively differentiated between high-risk and low-risk patients, with an AUC of 0.733 (Figure 4) for these factors. Sensitivity was 75.5%, specificity 63.1%, and the Youden index 0.386. A 16.7% threshold identified high-risk patients for sustained anemia, with an NPV of 95.4%. The model showed good calibration, with Hosmer–Lemeshow test P-values of 0.449, and internal validation via 1,000 bootstrap resamples confirmed its robustness, with a C-index of 0.732 (Figure 5). Furthermore, decision curve analysis indicated that the nomogram-based risk model offered a greater net clinical benefit across a broad spectrum of threshold probabilities compared to “treat-all” or “treat-none” strategies (see Figure 6).

A multivariate logistic regression analysis, adjusted for potential confounding variables, revealed that a postoperative reduction in hemoglobin concentration exceeding 20% was significantly associated with an elevated risk of cardiovascular events within 30 days, as indicated by an odds ratio (OR) of 2.187 and a 95% confidence interval (CI) of 1.070–4.470 (Table 4). Furthermore, both the maximum diameter of an abdominal aortic aneurysm (AAA) and the patient’s age were found to be correlated with the risk of experiencing cardiovascular events within 30 days following surgery.

**TABLE 4:** Multivariate logistic regression analysis of post-EVAR hemoglobin changes on 30-day cardiovascular events.

Variables	B	OR (95% CI)	p value
Age	0.054	1.056 (1.018–1.094)	0.003
Maximum diameter of abdominal aortic aneurysm,mm	0.032	0.420 (0.256–0.690)	0.001
Absence of PAD	−0.006	0.994 (0.227–4.357)	0.994
The decrease surpasses 20%	0.783	2.187 (1.070–4.470)	0.032

Sensitivity analyses in patients without RBC transfusions demonstrated that the increased risk of 30-day cardiovascular events was remained high for hemoglobin concentration exceeding 20% patients (OR 2.254; 95% CI 1.018 to 4.991,p = 0.045).

## Discussion

While the overall prognosis following endovascular abdominal aortic repair (EVAR) is generally positive, a subset of patients experiences a notable decline in hemoglobin (Hb) levels postoperatively. Recent studies ^12^indicate that Hb levels can decrease significantly within the first 24 h after EVAR, underscoring the necessity to investigate the pattern and timing of postoperative Hb changes to enable timely interventions. Currently, clinical guidelines lack a systematic description of the dynamic changes in Hb following EVAR and do not provide clear recommendations for intervention strategies or risk prediction models for Hb decline.

To our knowledge, this is the first study to examine the engagement trajectories in a hemoglobin change rate after endovascular aneurysm repair (EVAR). In this study, latent class trajectory analysis identified that approximately 16.8% of patients exhibited a decline in hemoglobin (Hb) levels following endovascular aneurysm repair (EVAR). The most significant reduction was observed within the first 48 h postoperatively, with an average decrease of 20%. In the persistent-decline group, patients exhibited a hemoglobin reduction of more than 20% postoperatively, indicating a sustained and clinically significant downward trend. From postoperative day 3 to day 5, Hb levels gradually stabilized. These results align with previous studies (Li et al., 2023; Valentijn et al., 2013), which also noted a continued decline in Hb levels throughout the first postoperative week.

Our study reveals that patients exhibiting persistent Hb decline were significantly older compared to those in the stable Hb group. Multivariate analysis further identified age as an independent predictor of Hb reduction (OR = 1.030; 95% CI: 1.007–1.054; P = 0.011). With advancing age, hematopoietic stem cell function progressively deteriorates, resulting in diminished erythropoietic capacity. Consequently, elderly patients may possess a limited ability to compensate for intraoperative blood loss or to recover from postoperative anemia. Furthermore, the age-associated decline in renal function may lead to reduced postoperative erythropoietin (EPO) production, thereby further impairing erythropoiesis. Elderly patients often present with a higher prevalence of comorbidities, which increases their susceptibility to intraoperative hemodynamic instability, impaired oxygen delivery, and tissue hypoxia. These factors collectively contribute to exacerbating hemoglobin (Hb) decline.

PAD patients exhibit a higher likelihood of experiencing a sustained decline in hemoglobin (Hb) levels following endovascular aneurysm repair (EVAR), as indicated by both preoperative factors (OR = 0.432; 95%CI: 0.188–0.953; P = 0.038). A potential explanation for the elevated incidence rate observed in this study may be attributed to the complexity and extended duration of surgical procedures required to revascularize occluded arteries in the lower limbs of our high-risk patient cohort, who are frequently affected by lower limb arterial occlusive disease. Previous research has documented a high prevalence of atherosclerotic comorbidities in patients with abdominal aortic aneurysms (AAA), including coronary artery disease (34.2%–43%) and peripheral arterial disease (19%–43.6%) (Adiarto et al., 2023; Hye et al., 2015). Persistent Hb decline is more frequently observed in patients with underlying atherosclerosis or CAD (Rymer and Rao, 2018),potentially due to pre-existing endothelial dysfunction and the chronic inflammatory state associated with atherosclerosis. These conditions may exacerbate the inflammatory response initiated by EVAR. Subsequent to endovascular aneurysm repair (EVAR), patients may experience a systemic inflammatory response termed post-implantation syndrome (PIS). This syndrome has the potential to diminish hemoglobin (Hb) levels by either suppressing erythropoiesis or expediting the destruction of red blood cells. Ongoing inflammation may further compromise bone marrow functionality and the production of erythroid cells, thereby resulting in a continuous decline in Hb levels (Li et al., 2023; Tsilimigras et al., 2018; Arnaoutoglou et al., 2015).

Our study revealed that an increased maximum diameter of an abdominal aortic aneurysm is significantly correlated with a heightened risk of persistent hemoglobin (Hb) decline following EVAR (preoperative factor: OR = 1.020; 95% CI: 1.005–1.036; P < 0.009). A larger aneurysm diameter generally indicates a larger aneurysm volume, extended operative duration, and intensified manipulation of the stent graft and catheters, which may lead to endothelial injury or rupture of small vessels, causing microbleeding during the procedure. Additionally, large aneurysms are often associated with intraluminal thrombus, calcification, and chronic inflammation. Postoperative sac decompression and blood volume redistribution may contribute to functional blood loss (Kim et al., 2023). Furthermore, a larger aneurysm diameter signifies more extensive structural wall damage and heightened local oxidative stress and inflammation, both of which may impair erythropoiesis or accelerate red blood cell destruction, thereby delaying Hb recovery. Moreover, aneurysm diameter is positively associated with the incidence of endoleak, where ongoing sac perfusion may result in occult blood loss and exacerbate anemia progression (Cannavale et al., 2020).

Iliac artery aneurysm presenting with bilateral involvement as a significant anatomical marker of disease extent and surgical complexity. Our findings indicate that iliac artery aneurysm presenting with bilateral involvement is significantly correlated with a heightened risk of postoperative hemoglobin (Hb) decline (postoperative factor: OR = 0.449; 95% CI: 0.280–0.718; P = 0.001). During the endovascular aneurysm repair (EVAR) of merging iliac artery aneurysms, the segmental elongation of the aneurysm typically necessitates more extensive internal graft coverage, prolonged operative time, and increased contact between the device and the vascular wall, all of which elevate the potential for endothelial damage and microvascular bleeding. Intraoperatively, the repeated manipulation of guidewires and catheters within elongated and tortuous vessels may elevate the risk of microvascular trauma and subclinical dissection, thereby exacerbating Hb decline. From a hemodynamic perspective, long-segment aneurysms can significantly influence sac perfusion patterns, thereby elevating the risk of endoleaks and undetected blood loss (Phan et al., 2015).

Despite EVAR being a minimally invasive technique, a certain level of intraoperative blood loss is frequently observed (Wang et al., 2005), particularly in patients with complex vascular anatomy or extended procedural durations. Blood loss during EVAR often occurs due to leakage during sheath and catheter exchanges, or as a result of procedural complications such as access-related injuries, aneurysm rupture, or device mismatch (Minion and Davenport, 2012). Navigating through stiff or calcified iliac arteries often necessitates repeated adjustments, during which brief sheath dislodgement or unstable guidewire positioning can result in continuous bleeding. The use of large-diameter sheaths may elevate the risk of bleeding at the femoral access site. Despite the application of closure devices, complications such as sealing failure, hematoma, or pseudoaneurysm formation may still occur. Montán et al. (2013) identified that open femoral access, the use of branched or unilateral iliac stent grafts, larger device profiles, and larger aneurysm diameters were all associated with increased perioperative blood loss (Montán et al., 2013). Furthermore, previous studies investigating closure devices and access site management indicate that different closure techniques can affect the risk of intra- and postoperative bleeding (Wang et al., 2023). For instance, heavily calcified femoral arteries may hinder the proper deployment of closure devices, whereas surgical cutdown provides greater control but increases soft tissue trauma. Longer procedures often indicate technical challenges, such as guidewire manipulation and stent deployment issues, and are linked to hypothermia, extended anesthesia, and increased inflammation, which can affect red blood cell production and destruction.

In this observational cohort of EVAR patients, we found that a postoperative hemoglobin decrease exceeding 20% was inversely related to 30-day cardiovascular events. To mitigate bias from RBC transfusions, we conducted sensitivity analyses excluding patients who received transfusions, which confirmed that hemoglobin decrease was still linked to a higher risk of 30-day postoperative cardiovascular events. This is the first study to assess the impact of a hemoglobin decrease over 20% on these events in EVAR patients. In patients who did not receive a blood transfusion, a postoperative decrease in hemoglobin (Hb) exceeding 20% constitutes an independent risk factor for cardiovascular events within 30 days, with the risk being 2.254 times greater compared to the group with an Hb decrease of 20% or less (95% CI: 1.018–4.991, P = 0.045). Few studies have evaluated the risk of Hb decrease on postoperative outcomes for EVAR patients. Our results are consistent with several other studies in which low postoperative Hb concentrations were associated with a worse outcome (Li et al., 2023; Valentijn et al., 2013). However, this study does not determine whether these patients should be treated or identify the best treatment option.

This study offers a straightforward, customized approach for predicting sustained hemoglobin decline after EVAR, in the form of a nomogram. Clinically, this scoring system aids in effective preoperative risk stratification, with scores above 70 points indicating over a 90% probability of sustained hemoglobin decline.

## Limitations

This study has several limitations. First, this study included an observational cohort of EVAR patients. Patient data, including Hb concentrations, RBC transfusions and study endpoints, were recorded retrospectively, so causality between Hb concentration, RBC transfusions and outcome cannot be established. Second, patients who required postoperative transfusion were excluded to avoid confounding effects on hemoglobin dynamics. Although this allowed us to observe the natural perioperative hemoglobin trajectory without interference from major bleeding events, it inevitably excluded patients with the most clinically significant hemoglobin declines. Therefore, the generalizability of our findings to all EVAR patients—particularly those with postoperative bleeding requiring transfusion—may be limited. Results of the present study may not be applicable to other patient groups. Third, as this was an observational study, further prospective studies are needed to confirm and extend our findings.

## Conclusion

In conclusion, our study determined that advanced age, increased maximum diameter of abdominal aortic aneurysm, and the presence of bilateral iliac artery aneurysms are associated with a sustained decline in hemoglobin levels following endovascular aneurysm repair (EVAR). Furthermore, a reduction in hemoglobin concentration exceeding 20% after elective EVAR surgery serves as a strong predictor of cardiovascular events within the subsequent 30 days.

## Data Availability

The raw data supporting the conclusions of this article will be made available by the authors, without undue reservation.
